# Evolution of plant δ^1^-pyrroline-5-carboxylate reductases from phylogenetic and structural perspectives

**DOI:** 10.3389/fpls.2015.00567

**Published:** 2015-08-03

**Authors:** Giuseppe Forlani, Kira S. Makarova, Milosz Ruszkowski, Michele Bertazzini, Boguslaw Nocek

**Affiliations:** ^1^Department of Life Science and Biotechnology, University of FerraraFerrara, Italy; ^2^National Center for Biotechnology Information, National Library of Medicine, National Institutes of Health, BethesdaMD, USA; ^3^Synchrotron Radiation Research Section, Macromolecular Crystallography Laboratory, National Cancer Institute, Argonne National Laboratory, ArgonneIL, USA; ^4^The Bioscience Division, Argonne National Laboratory, ArgonneIL, USA

**Keywords:** P5C reductase, phylogenetic analysis, 3-D structures of P5CRs, oligomer structure prediction, cofactor preference

## Abstract

Proline plays a crucial role in cell growth and stress responses, and its accumulation is essential for the tolerance of adverse environmental conditions in plants. Two routes are used to biosynthesize proline in plants. The main route uses glutamate as a precursor, while in the other route proline is derived from ornithine. The terminal step of both pathways, the conversion of δ^1^-pyrroline-5-carboxylate (P5C) to L-proline, is catalyzed by P5C reductase (P5CR) using NADH or NADPH as a cofactor. Since P5CRs are important housekeeping enzymes, they are conserved across all domains of life and appear to be relatively unaffected throughout evolution. However, global analysis of these enzymes unveiled significant functional diversity in the preference for cofactors (NADPH vs. NADH), variation in metal dependence and the differences in the oligomeric state. In our study we investigated evolutionary patterns through phylogenetic and structural analysis of P5CR representatives from all kingdoms of life, with emphasis on the plant species. We also attempted to correlate local sequence/structure variation among the functionally and structurally characterized members of the family.

## Introduction

L-proline is a unique multifunctional amino acid that is increasingly being associated with many important aspects of biology (Nocek et al., 2005; Szabados and Savouré, 2010). Its cyclic side chain restricts the conformational flexibility of the backbone in the protein structure. Furthermore, proline shows geometric *cis–trans* isomerism, a phenomenon that plays a central role in the folding and function of proteins (Morgan and Rubenstein, 2013). Repetitive proline-rich sequences are found in many proteins, and in several cases they are believed to be signaling elements (Kay et al., 2000). Besides its structural role as a component of proteins, proline accumulation represents one of the major strategies used by plants as a response to various abiotic and biotic stress conditions (Lehmann et al., 2010; Szabados and Savouré, 2010; Funck et al., 2012). Typically, the accumulation occurs in the cytoplasm where it may also function as a molecular chaperon stabilizing the structure of proteins and buffering cellular redox potential (Maggio et al., 2002). Proline synthesis is directly linked to the NAD(P)H/NAD(P)^+^ redox pair, indicating that it might play a secondary role as a redox shuttle, used to transfer redox equivalents between mitochondria and the cytosol (Poolman et al., 1983; Phang, 1985). It was suggested that the cellular levels of proline are regulated by the rate of both synthesis and degradation. Due to the separation of these processes between cytoplasm and mitochondria, regulation of the intracellular proline transport is also possible (Lehmann et al., 2010).

Proline biosynthesis occurs via two routes: the glutamate and the ornithine pathway (Smith et al., 1980). The glutamate pathway is the primary route for proline biosynthesis in bacteria, whereas in eukaryotes it is predominantly used under stress and limited nitrogen availability. Higher plants use the pathway from ornithine, as the main route under normal conditions (Delauney and Verma, 1993). Four reaction steps, catalyzed by three enzymes are required to convert glutamate to proline. In the first step, glutamate is phosphorylated by γ-glutamyl kinase (EC 2.7.2.11) yielding γ-glutamyl phosphate. In the second step, γ-glutamyl phosphate is converted by the enzyme γ-glutamyl phosphate reductase (EC 1.2.1.41) to glutamate γ-semialdehyde. In plants a single bifunctional enzyme, namely P5C synthetase, catalyzes both reactions. Glutamate γ-semialdehyde undergoes a spontaneous cyclization to δ^1^-pyrroline-5-carboxylate (P5C). In the terminal step, that is catalyzed by P5C reductase (P5CR; EC 1.5.1.2.), P5C is reduced by the cofactor NAD(P)H to yield L-proline and the oxidized cofactor NAD(P)^+^. The enzymes ornithine amino transferase (EC 2.6.1.13), and P5CR are required for the biosynthesis of proline from ornithine. Both pathways share the last enzymatic step, catalyzed by P5CR. This terminal step appears to be essential in some organisms such as *Arabidopsis thaliana*, where deletion of the *P5CR* gene was reported to be embryo-lethal (Funck et al., 2012). Similarly in fungi, the inhibition of the *P5CR* gene expression or activity leads to drastically reduced pathogenicity (Adachi et al., 2004). Also, specific inhibitors of P5CR exert cytotoxic effects, and could be potentially exploited for herbicide (Forlani et al., 2008) and antibiotic (Forlani et al., 2012) design. It was postulated that the enzymatic activity of P5CR is regulated in various plant tissues at different developmental stages. In young, metabolically active tissues proline likely functions as an energy and/or nitrogen and carbon source, while it is mainly related to dehydration in mature tissues (Hua et al., 1997).

The P5CRs constitute a very interesting and large family of enzymes (over 37,000 representatives in the NCBI database), which in addition to their elementary cellular role, appear to be involved in many other biological functions. Even though proline metabolism has been studied for over 40 years, this important family remained enigmatic due to the lack of three-dimensional structures. In recent years several structures of bacterial and mammalian P5CRs have been determined. However, only a handful were analyzed and published. As a consequence, there is still a significant knowledge gap especially for plant representatives, which have not been structurally characterized to date. In order to address this problem, and improve understanding of these important enzymes, we analyzed sequences of plant P5CRs and correlated them with currently available structural information. Analysis of evolutionary origin and comparison of sequences and structures of known representatives reveal a number of important structural features, which indicate a global trend, for the plant P5CRs and the entire family.

## Materials and Methods

### Sequence Analysis

The sequences of P5CR proteins from plants and algae were retrieved from *Refseq* database (Pruitt et al., 2014; as of February 2015) as a result of PSIBLAST search (Altschul et al., 1997; one iteration with the *e*-value cutoff of 0.01, and without either low complexity filtering or composition-based statistics). The additional eukaryotic sequences were retrieved from OrthoMCL database (Fischer et al., 2011; http://www.orthomcl.org/orthomcl/), the orthology group OG5_126801. Archaeal and bacterial sequences were retrieved from COG database for COG0345. The BLAST-Clust program (Wheeler and Bhagwat, 2007) set up with the length coverage cutoff of 0.95 and the score coverage threshold (bit score divided by alignment length) of 1.5 was used for clustering archaeal and bacterial sequences (Supplementary Material). One sequence was chosen for each cluster for further analysis. Multiple alignments were built for eukaryotic and prokaryotic sequences using MUSCLE program (Edgar, 2004). A few incomplete sequences were discarded, resulting in a set of 728 aligned protein sequences. The alignment was filtered to exclude sites with gap character fraction >0.5 and homogeneity <0.1 (Yutin et al., 2008). The resulting 298 informative positions of the alignment were used for maximum likelihood tree reconstruction using the *FastTree* program (Price et al., 2010) with default parameters: JTT evolutionary model, discrete gamma model with 20 rate categories. The *FastTree* software was also used to compute bootstrap values.

### Cloning, Overexpression, and Purification of *At*P5CR and *Bc*P5CR

The coding genes of *Arabidopsis thaliana* P5CR (*At*P5CR; Giberti et al., 2014) and *Bacillus cereus* P5CR (*Bc*P5CR, Bc_2977; Q81C08) were cloned into vector pMCSG68 according to the standard protocol described previously (Eschenfeldt et al., 2013; Nocek et al., 2014). The pMCSG68 vector introduces a His_6_-tag followed by the Tobacco Etch Virus (TEV) protease cleavage site at the N-terminus of the expressed protein. The correctness of the insert was confirmed by DNA sequencing. Overexpression was carried out in BL21 Gold *E. coli* cells (Agilent Technologies). The bacteria were cultured with shaking at 210 rpm in LB medium supplemented with 150 μg/ml ampicillin at 37°C until the OD_600_ reached 1.0. The temperature was lowered to 18°C and isopropyl-D-thiogalactopyranoside (IPTG) was added to a final concentration of 0.5 mM. The culture was grown for 18 h and then centrifuged at 4500 rpm for 10 min at 4°C. Cell pellet from 1 L culture was resuspended in 35 mL of lysis buffer (50 mM HEPES sodium salt pH 8.0, 500 mM NaCl, 5% glycerol, 20 mM imidazole, 10 mM β-mercaptoethanol) and stored at -80°C. The samples were thawed and the cells were disrupted by sonication using bursts of total duration of 5 min, with appropriate intervals for cooling. Cell debris was pelleted by centrifugation at 15 000 rpm for 30 min at 4°C. The supernatant was applied to a column packed with 10 mL of HisTrap HP resin (GE Healthcare), connected to VacMan (Promega) and the chromatographic process was accelerated with a vacuum pump (developed by R. Jedrzejczak). The column was washed with 20 bed volumes of lysis buffer and the His_6_-tagged P5CRs were eluted with 25 mL of elution buffer (50 mM HEPES pH 8.0, 500 mM NaCl; 500 mM imidazole; 2 mM DTT). The His_6_-tag was cleaved with TEV protease (2 mg of a His_6_-tagged form) overnight at 4°C and dialysis to remove the excess of imidazole was carried out simultaneously. The resulting solution was mixed with His-Trap HP resin to capture the cleaved His_6_-tag and the His_6_-tagged TEV protease and the flow through containing the protein of interest was collected and concentrated.

### Determination of Molecular Weight of P5CRs (*At*P5CR and *Bc*P5CR)

The molecular weights of P5CR proteins were evaluated according to previously described protocol (Nocek et al., 2005), by size exclusion chromatography (SEC) on a HiLoad 16/600 Superdex 200 Prep Grade column (GE Healthcare). 1.5 mL aliquots of purified and concentrated proteins (∼5mg/mL) were centrifuged for 5 min prior to the injection onto the column, which was equilibrated and run in lysis buffer. The column was calibrated with chymotrypsynogen A (25 kDa); albumin (67 kDa), *Streptococcus pyogenes* (*Sp*P5CR, 275 kDa decamer confirmed by SEC and X-ray crystallography methods; Nocek et al., 2005), and thyroglobulin (669 kDa) as standards. The calibration curve of *K*_av_ versus log molecular weight was prepared by using the equation *K*_av_= *V*_e_ – *V*_o_/*V*_t_ – *V*_o_, where *V*_e_ = elution volume for the protein, *V*_o_ = column void volume, and *V*_t_ = total bed volume.

### 3-D Structures of P5CRs

A search in the Protein Data Bank archive revealed eleven models of six unique proteins representing P5CRs from the following organisms: *Homo sapiens* (PDB id: 2GER, 2GR9, 2GRA, 2IZZ- used for the most of analyses due to the highest resolution); *Bacillus cereus* PDBid: 3GT0 (NCBI taxonomy ID 226900); *Coxiella burnetii* PDBid: 3TRI (NCBI taxonomy ID 227377); *Plasmodium falciparum* PDBid: 2RCY (NCBI taxonomy ID 36329); *Streptococcus pyogenes* PDBid: 2AHR, 2AMF (NCBI taxonomy ID 301447); *Neisseria meningitides* PDBid: 1YQG, 2AG8 (NCBI taxonomy ID 122586). During the preparation of this manuscript we were able to determine a low-resolution (3.40 Å) structure of rice *Oryza sativa* P5CR (*Os*P5CR; Forlani et al., 2015), and used some of the relevant information to better correlate sequence to structural features.

## Results and Discussion

### Sequence and Phylogenetic Analysis of P5CR Family

P5CRs (common synonyms: 1-pyrroline-5-carboxylate reductase, δ^1^-pyrroline-5-carboxylate reductase, P5C reductase) are important housekeeping enzymes that are broadly distributed across all three domains of life. According to estimates from the OrthoMCL database (Fischer et al., 2011) they were identified in ∼80% of archaea and bacteria and in 88% of eukaryotes in the orthology group: OG5_126801. Typical plant P5CRs are composed of two domains [NAD(P)-binding Rossmann-like domain, CATH 3.40.50.720; and ProC C-terminal domain, CATH 1.10.3730.10] and are ∼280 amino acid (a.a.) long. For example, *At*P5CR has 274 a.a., *Os*P5CR has 284 a.a., while the longest P5CR (347 a.a. long) is found in the green alga *Chlamydomonas reinhardtii*. They can be recognized by the Prosite PA line pattern sequence signature [PALF]-x(2,3)-[LIV]-x(3)-[LIVM]-[STAC]-[STV]-x-[GANK]-G-x-T-x(2)-[AG]-[LIV]-x(2)-[LMF]-[DENQK] (Sigrist et al., 2013). Most of the sequences with this signature are likely *bona fide* P5CRs.

**FIGURE 1:**
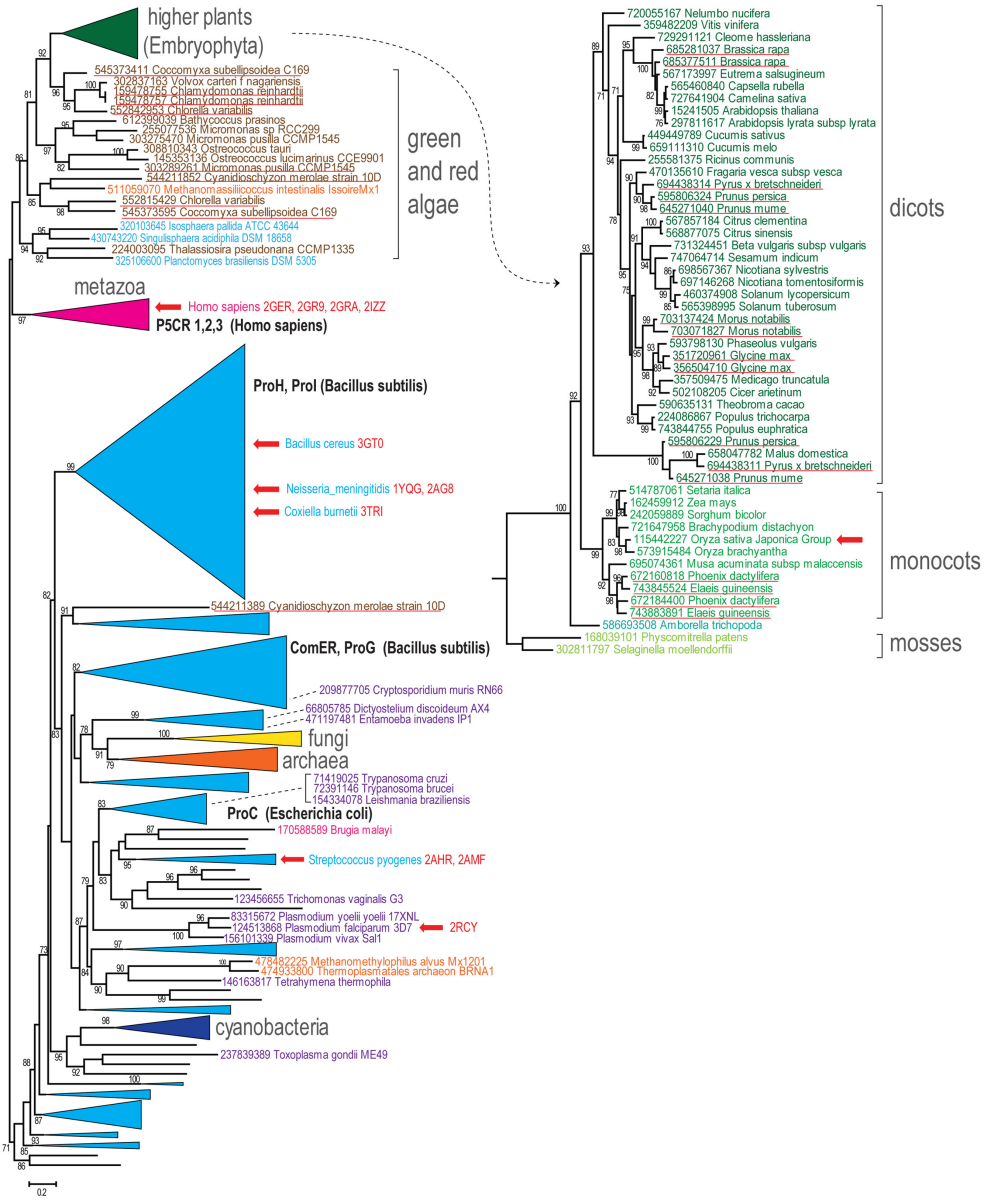
**Phylogenetic tree of predicted members of the pyrroline-5-carboxylate reductase (P5CR) family.** Maximum-likelihood phylogenetic unrooted tree was built with the FastTree program using a multiple alignment for 728 P5CR amino acid sequences built by MUSCLE. FastTree program was also used to compute bootstrap values; only values >70% are indicated. GenBank identifier number and systematic organism name marked the terminal nodes of the tree. Several bacterial terminal nodes are hidden for clarity and can be viewed in the Supplementary Material with complete tree data (Supplementary Material File 1). Several branches are collapsed and shown as triangles denoted by the respective lineage name. The branch corresponding to the higher plants is expanded and shown separately on the right side. Color code: bacteria, light blue; cyanobacteria, dark blue; archaea, orange; metazoan, purple; other eukaryotes, dark purple; fungi, yellow; plants, green shades; algae, dark brown. The characterized P5CR sequences are indicated by respective gene name highlighted by bold (for those that are within collapsed branches the organism name is also indicated). The organism names and PDB code are provided for P5CR proteins with solved crystallographic structure and red arrows show their location in the tree. Duplications in plants and algae are underlined.

Sequences of P5CR from all three domains of life were evaluated to reveal their evolutionary relationships, with the purpose of gaining more insights into evolution of plant orthologs. A detailed analysis of a very large group of proteins from the P5CR family (728 sequences), in which we included the most current and complete set of representatives from higher plants and algae, yielded the tree shown in **Figure 1**. The general topology of the tree is in agreement with a phylogenic analysis, which has been recently published based on a small subset of P5CRs (Fichman et al., 2014). In both analyses, metazoa are grouped with plants and algae apart from a large branch that includes most of bacteria, archaea, and other eukaryotic species. Remarkably, none of the three domains of life are monophyletic (i.e., they do not share a common ancestor). Majority of bacterial and archaeal lineages are not monophyletic either, with exception of cyanobacteria and *Deinococcus–Thermus* lineages, which are mostly monophyletic (**Figure 1** and Supplementary Material File 1). A largely monophyletic clade of fungi and the remaining eukaryotes from different taxonomic groups are scattered among bacterial branches (**Figure 1**).

Since many of these species possess only one P5CR gene (or have lineage-specific duplications), the lack of clear concordance with organismal taxonomy could be explained by xenologous gene displacement (displacement of the ancestral gene by a horizontal gene transfer; Koonin et al., 2001). In several bacteria and archaea, however, there are distant and relatively fast-evolving paralogs of P5CR (such as ProG and ComER in *Bacillus subtilis*). These diverged paralogs are mostly grouped together (Supplementary Material File 1). As reported previously (Fichman et al., 2014), they are unlikely involved in proline biosynthesis and thus represent potential examples of subfunctionalization (Lynch and Force, 2000). It is noteworthy that there is at least one duplicate, which generally corresponds to mitochondrial pyrroline-5-carboxylate reductase 1 (P5CR1) and pyrroline-5-carboxylate reductase 3 (P5CR3) from *Homo sapiens*, that could be dated back to the metazoan ancestor (Fichman et al., 2014; Supplementary Material).

Genes from higher plants and algae are monophyletic and the respective branch is mostly concordant with organismal taxonomy (**Figure 1**). Although most algae have at least two genes for P5CR, higher plants seem to inherit only one gene. A few lineage-specific duplications in higher plants, such as *Glycine max*, have been reported before (Shultz et al., 2006), but now many more cases of P5CR gene duplication can be observed (**Figure 1**, underlined in red). Some of them occurred relatively recently in narrow lineages, such as *Brassica* and *Morus* genera, but other examples include duplications that occurred earlier in evolution, at least in the common ancestor of *Arecales* (*Elaeis* and *Phoenix* genera) and in the common ancestor of *Maloideae* lineage (*Pyrus, Malus*, and *Prunus* genera). The latter case is quite notable, since it seems that the duplication was followed by an acceleration of the evolutionary rate. This fast evolution could have caused an erroneous placement of the *Maloideae* branch at the bottom of dicot branch. It could be expected that in these species P5CR gene underwent subfunctionalization. The generally accepted endosymbiosis transfer theory links chloroplasts found in plants and eukaryotic algae to cyanobacteria. However, our analysis does not reveal any evidence that P5CR genes in plants were acquired from cyanobacteria, which is an agreement with the results published before (Cullis et al., 2009; Fichman et al., 2014; **Figure 1**).

Thus, it appears that vertical descent dominates in evolution of P5CR genes in higher plants and algae. Some enzymes are able to function in both chloroplast and cytoplasm, whereas relatively rare cases of duplications might be related to differentiation of targeting affinities of the paralogs and are an interesting subject for further experimental study.

### A Minimalist P5CR Structure

**FIGURE 2:**
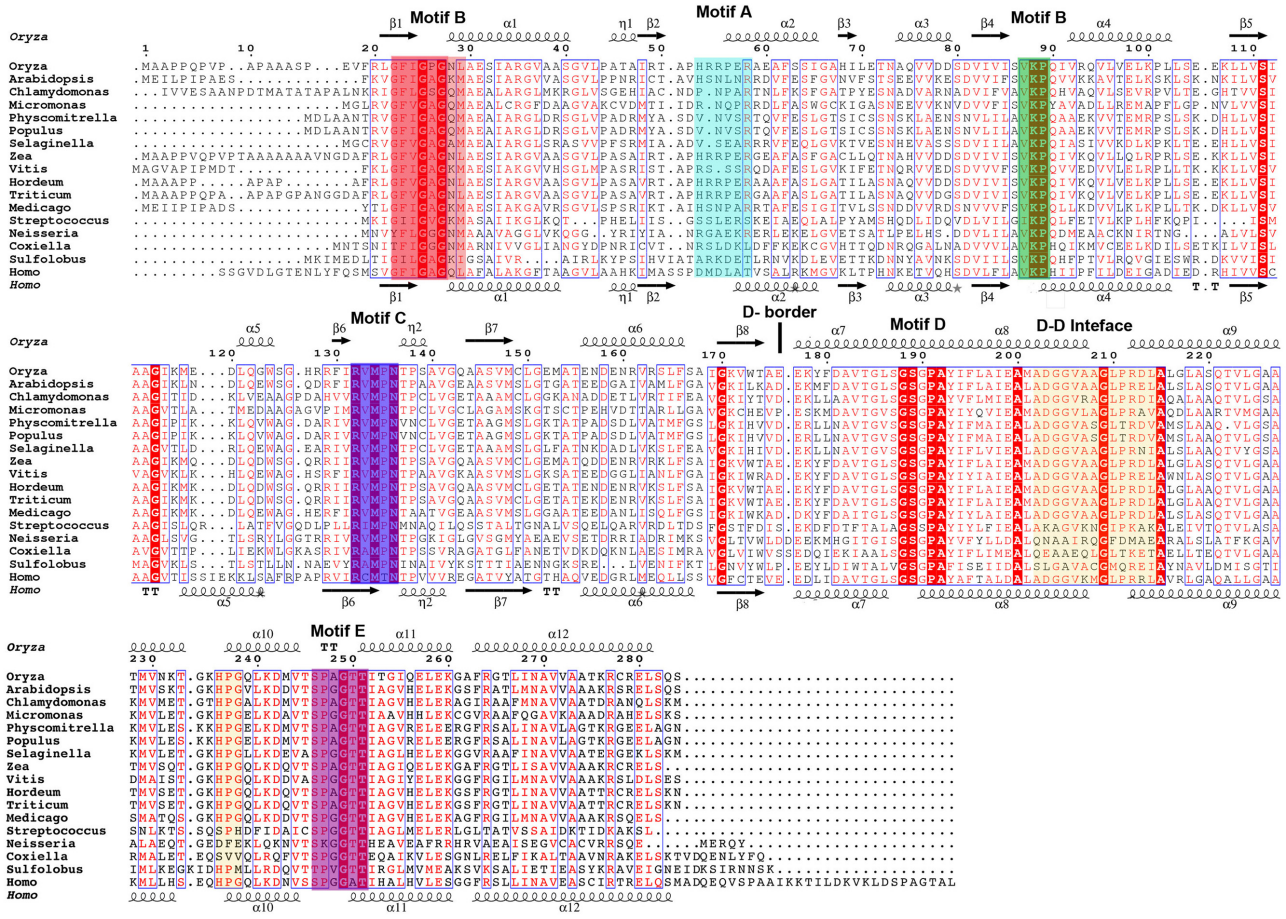
**Multiple sequence alignment of selected representatives of the P5CR family.** For simplicity, 17 sequences of characterized and uncharacterized representatives of P5CRs have been aligned using Clustal W2 (Larkin et al., 2007) and Espript 3.0 (Robert and Gouet, 2014). Sequence identities are highlighted in red and similarities are displayed as red letters. The corresponding secondary structures of plant *Os*P5CR and human *Hs*P5CR are shown on the top and the bottom (in black), respectively. Helices (α-helix; η-3_10_ helix) appear as scribble, beta strands (β-strand) as arrows. Conserved fingerprint motifs are highlighted in color and labeled (Motif A, cyan; Motif B, red and green; Motif C, blue; Motif E, magenta; D–D, dimer–dimer interfaces in orange; D-border, border of domains). The following sequences were used, with the accession numbers and PDBid indicated in parentheses: *Homo sapiens* (P32322; 2GER); *Sulfolobus solfataricus* (Q97ZT3); *Neisseria meningitides* (Q9K1N1; 1YQG); *Streptococcus pyogenes* M1 GAS (Q9A1S9; 2AHR); *Medicago truncatula* (gi| 357509475); *Triticum aestivum* (gi| 58843559); *Hordeum vulgare (*gi| 326512934); *Vitis vinifera (*gi| 359482209); *Zea mays* (gi| 162459912); *Selaginella moellendorffii* (gi| 302811968); *Populus trichocarpa* (gi| 224086867); *Physcomitrella patens* (XP_001772037.1); *Micromonas* sp. *RCC299* (gi| 255077536); *Chlamydomonas reinhardtii* (gi| 159478755, the first 60 residues were not aligned); *Arabidopsis thaliana* (NP_196984.1); *Oryza sativa* ssp. *japonica* (gi| 215695199).

Pyrroline-5-carboxylate reductase enzymes characterized to date are composed of two unique domains: an N-terminal, dinucleotide binding domain (residues 1–175 in *Os*P5CR), and a C-terminal domain (residues 176–284 in *Os*P5CR, **Figure 2**).

The typical N-terminal domain is comprised of a central core formed by parallel β-sheet (β3, β2, β1, β4, β5, β6) surrounded by six alpha helices (α1-α6) and two 3_10_ helices (η1, η2) in the topological order (β1, α1, η1, β2, α2, β3, α3, β4, α4, β5, α5, β6, η2, β7, α6, β8), (Nocek et al., 2005; Meng et al., 2006). This domain is classified as a member of Pfam family PF03807 (F420_oxidored), and it is common amongst FAD or NAD(P)H dependent oxidoreductases (Carugo and Argos, 1997; Dym and Eisenberg, 2001; Kleiger and Eisenberg, 2002; Nocek et al., 2002). This fold is composed of three parallel β-strands linked by two α-helices, founding the so-called Rossmann fold (Rossmann et al., 1974). The Rossmann fold can be identified by the short amino acid sequence motif (G-x-x-G-x-G, Motif B; **Figure 2**), which binds one nucleotide cofactor molecule. Since the NAD(P)H molecule has two nucleotides (adenosine and nicotinamide riboside), NAD(P)H dependent oxidoreductases, such as P5CR enzymes, use a 3-layer sandwich fold which consists of two fused mononucleotide-binding motifs. These motifs are structurally related by a pseudo-twofold rotation within one domain, yet they do not share any similarity in the sequence (**Figure 2**; Bottoms et al., 2002).

**FIGURE 3:**
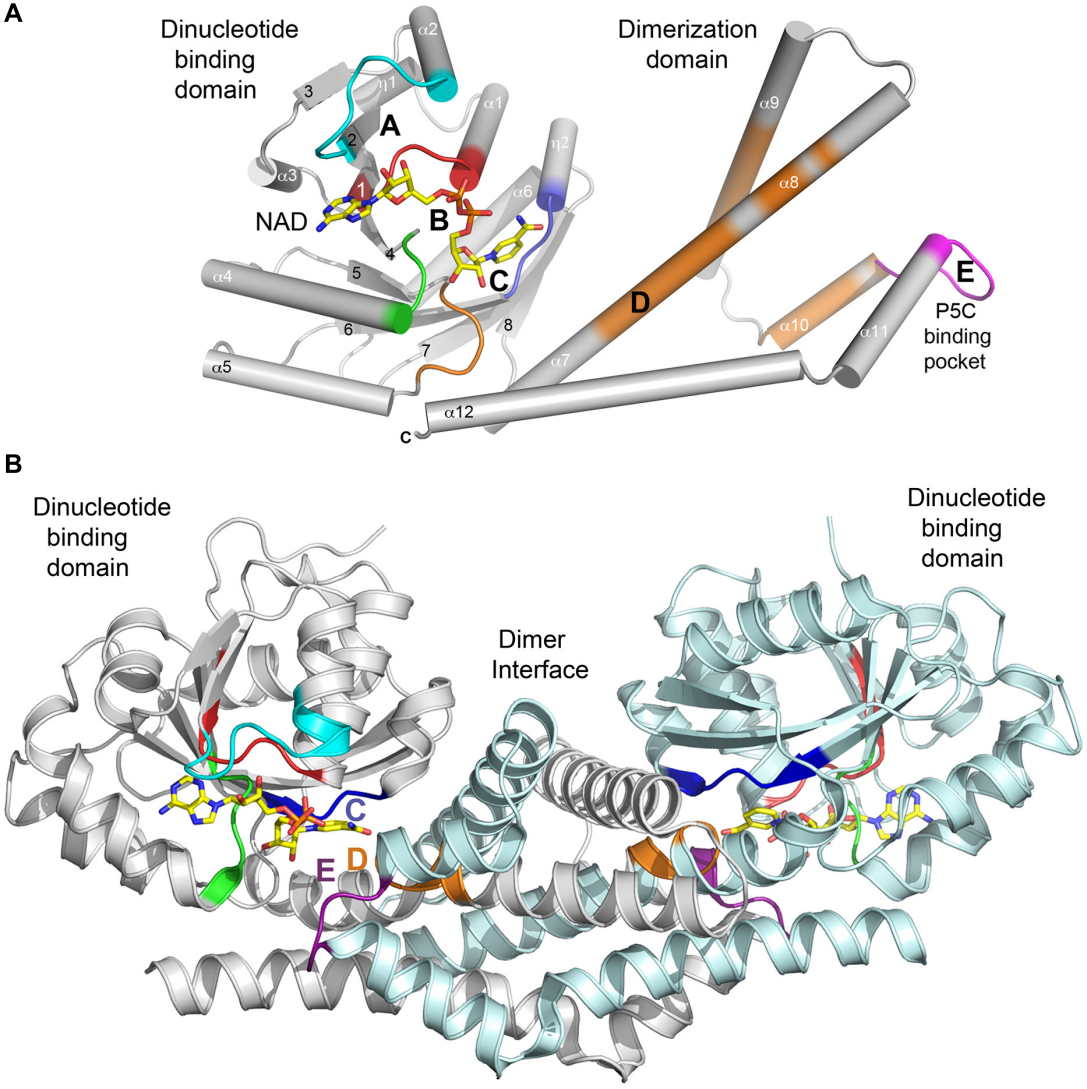
**Secondary structure of a typical P5CR enzyme. (A)** A diagram showing architecture of monomer of *Hs*P5CR (PDB id: 2IZZ). Parts of protein interacting with NAD are colored in cyan (A), red and green (B) and blue (C), while substrate (P5C, 1-pyrroline-5-carboxylic acid) pocket is colored in magenta (E). In addition, regions with highly conserved sequence are highlighted in orange. Secondary structure elements are labeled with numbers. **(B)** A dimer architecture of *Hs*P5CR, revealing the basic biological unit utilized by the P5CR family showing a proper active site arrangement with motifs C, E, and D (the hinge region motif) positioned closely.

The C-terminal domain is entirely alpha helical (six helices: α7–α12 in *Sp*P5CR) in typical size proteins such as plant P5CRs (**Figure 2**), while longer representatives such as *Homo sapiens* P5CR (*Hs*P5CR) have extended C-terminus, which was missing in the structure (Meng et al., 2006; Pfam family: PF14748). The C-terminal domains are involved in the homodimer formation and arrangement of the active site. Based on structures, it is evident that dimerization is essential to the formation of a completely functional enzyme, indicating that the dimer is a basic biological unit utilized by the P5CR family. To date, no monomeric structures of P5CR were reported, and only dimers (Nocek et al., 2005) or higher oligomers with even number of subunits were observed (Nocek et al., 2005; Meng et al., 2006). Also, chemical treatment of *Hs*P5CR decamer with 1–4 M urea showed that it dissociates into homodimers, and no monomeric forms were observed (Meng et al., 2006). The requirement for a dimeric configuration is unambiguous based on the analysis of the structures of a typical P5CR monomer (**Figures 3A,B**). In the monomer, two parts of the active site are divided and separated by ∼30 Å, creating what would be an inactive enzyme. In contrast, in the dimer the fully functional active sites are assembled through contribution from the N-terminal domain of one subunit of the dimer and the dimerization domain of the opposite subunit, and *vice-versa*. This way, two well-defined active sites are formed, within ∼13 Å distance between motif C of one subunit and motif E of the opposite subunit (**Figures 2 and 3B**).

### Dimer Interface

Tight packing of hook-like dimerization domains between two neighboring molecules and swapping of the C-terminal helices (α9–α11) makes the intertwined dimer interface. A three-dimensional domain swapping, in which a section of the monomeric protein is replaced by the matching part from a second monomer is one of the common structural adaptations used for protein oligomerization (Jones and Thornton, 1996; Bennett and Eisenberg, 2004). As there is no experimental evidence of reversible swapping of the domains (between monomer and dimer), it suggests that intertwined P5CR dimers might have evolved very early in evolution and became conserved. Sequence alignment analysis seems to support this hypothesis as almost complete conservation of the sequence for the first three helices of the C-terminal domain (residues 180–218 in *Os*P5CR) is observed in plant P5CRs, while ∼50% of sequence identity (as estimated based on the alignment) is observed for all other representatives annotated as P5CR in Pfam database (PF14748.1). In fact, these three-conserved helices form a core that is sufficient to produce a stable dimer on its own, as shown in the structure of *Bacillus cereus* P5CR (*Bc*P5CR, PDB id: 3GT0). The *Bc*P5CR structure model has residues 1–217 and is missing the last 55 residues (a full-length gene product of Bc_2977 has 272 residues), and yet it forms a stable dimeric structure. A hallmark of the dimerization core is a loop with a sequence motif G-S-x-P-A (Motif D, **Figure 2**), which is predicted to be a hinge region according to both *Hingeprot* and *StoneHinge* softwares (Emekli et al., 2008; Keating et al., 2009). This loop contributes to the formation of the active site pocket together with residues from the motifs C and E. It also stabilizes the active site core by a hydrogen bond formed between the conserved serine of the motif D with the last threonine of the motif E (S176, T238 in *Hs*P5CR; S189, T251in *Os*P5CR).

### P5CR Decamers

Examination of reported molecular weights (*M*_W_) of P5CR enzymes reveals the presence of several multimeric forms. These were estimated, based on the SEC experiments under non-denaturating conditions and were ranging from dimers (Nocek et al., 2005), octamers (Kenklies et al., 1999), decamers (Deutch et al., 1982; Nocek et al., 2005), and dodecamers (Murahama et al., 2001; Giberti et al., 2014) to even higher polymers (Krueger et al., 1986; Meng et al., 2009). However, results obtained for full-length members of P5CR superfamily by crystallographic studies consistently showed two oligomeric forms: dimeric and decameric (Nocek et al., 2005; Meng et al., 2006). Despite the different types of crystallization conditions, crystal forms, and variation in the sequence, the decamer is the highest oligomeric species observed by crystallography [especially in eukaryotic representatives: *Hs*P5CR (Meng et al., 2006), *Os*P5CR (Forlani et al., 2015) and *At*P5CR (see below)].

Even though the SEC technique is a powerful method for size fractionation of biomolecules, it is highly dependent on the accurate and precise calibration curve, temperature and several other factors, which may cause an error of up to 10% in the estimated molecular weight (Folta-Stogniew and Williams, 1999; Moreira et al., 2007). Considering 10% error, the forms estimated to be octamers and dodecamers could in fact be a decamers. In order to improve our SEC column calibration method, a representative of P5CRs from *Sp*P5CR was utilized. *Sp*P5CR has been experimentally characterized to be decamer by both SEC and X-ray crystallography methods and was used as one of the molecular weight markers (Nocek et al., 2005). Using this approach we were able to show that *At*P5CR, recently reported to be a dodecamer (Giberti et al., 2014), under our experimental conditions is more likely a decamer with an estimated molecular weight of 275 kDa (Supplementary Figure S1, 276 residues, theoretical *M*w deduced from the sequence 28,624 kDa and theoretical decamer *M*w = 286,240 kDa).

**FIGURE 4:**
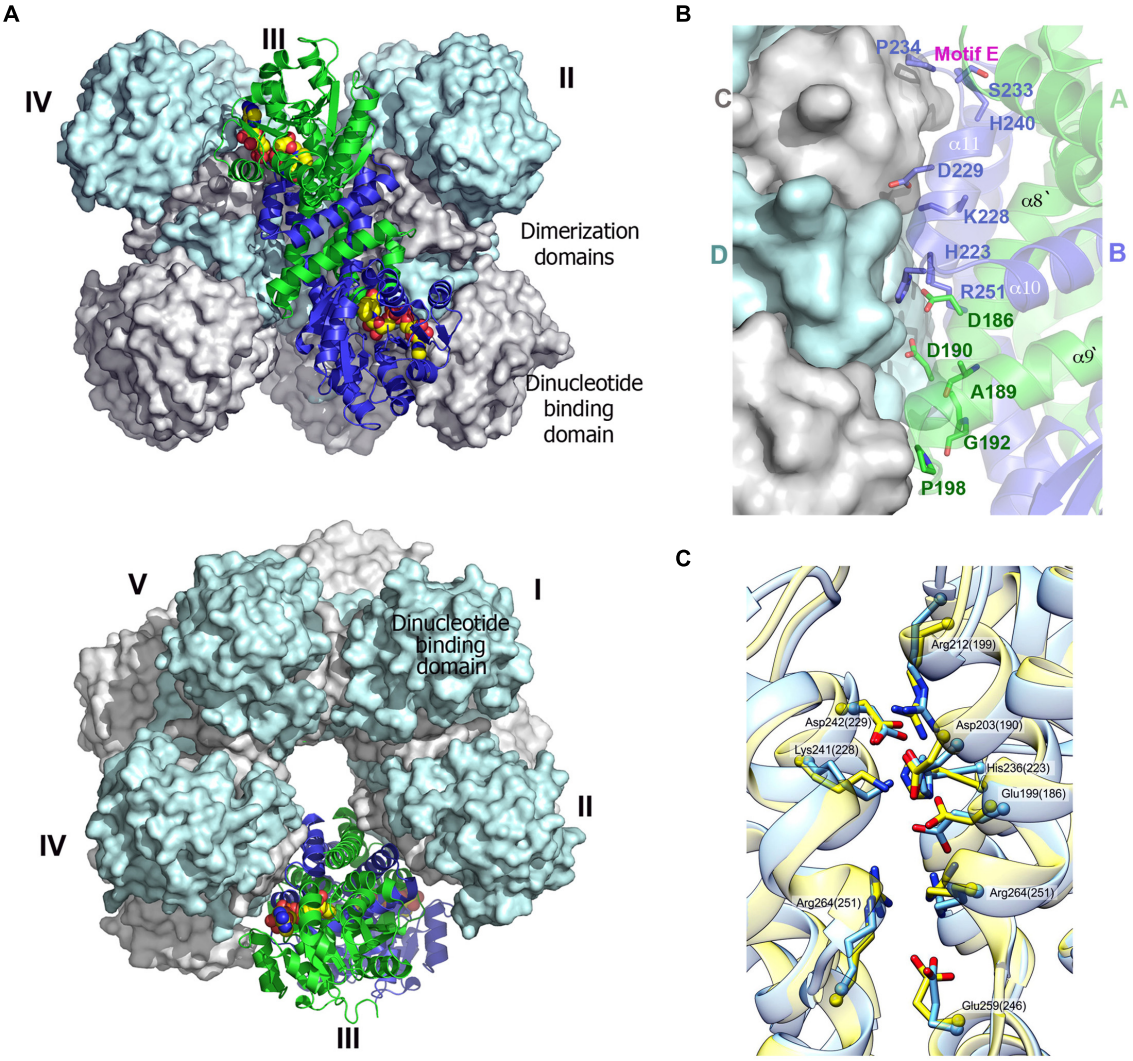
**Decameric structure of human P5CR. (A)** Two views of the decamer of *Hs*P5CR (PDB id: 2IZZ) related by 90° rotation, displaying five dimers (numbered) arranged around the fivefold symmetry axis (surfaces of four dimers are shown in gray and cyan, while one dimer is show as cartoon in green and blue. The NAD molecules located on the side of the dinucleotide binding domains are shown as a yellow space filled models. **(B)** Close-up view of the dimer–dimer interface, revealing positions of proposed key residues involved in decamer interface formation. **(C)** The dimer–dimer interface network pattern appears to be similar and conserved between *Hs*P5CR (yellow, the residues numbers in the brackets) and *Os*P5CR (blue).

A typical decameric structure of P5CR is described based on the structures of *Hs*P5CR (Meng et al., 2006) and *Os*P5CR representatives (Forlani et al., 2015). It resembles an hourglass-shaped assembly, which is formed by five closely interacting dimers, arranged around the fivefold symmetry axis. Monomers in each dimer are related by the twofold symmetry axis, and form a dimerization interface. The interacting dimerization domains form a very tight five-membered ring with a 25 Å opening in the center, while the dinucleotide-binding domains are located on the top and the bottom of the ring, and do not interact with each other (**Figure 4**). In contrast to the strong interactions observed in the dimerization interface of *Hs*P5CR and *Os*P5CR, the dimer–dimer interfaces forming the decamer are much weaker. In fact, analysis of these interactions using server PISA (Krissinel and Henrick, 2007) in *Hs*P5CR shows that the dimer interface is contributed by all helices from the C-terminus and buries about 4100 Å^2^ of the accessible surface area per dimer (which corresponds to about 25% of each subunit surface area). In contrast, the decamer interface is formed only by lateral interactions of the C-terminal α8–α12 (residues located between 196–214 and 235–260) and its complementary symmetry mates. This leads to a substantially smaller interface between four contributing elements (∼1100 Å^2^) from consecutive dimers, which accounts for less than 5% of each dimer surface (**Figure 4B**). Two pairs of loops from the dimers [from the top: the loop α8–α9 of molecule A and the loop formed between α10–α11 (Motif D) of molecule D; and from the bottom, the loop α8–α9 of molecule C and α10–α11 molecule B (Motif E, the active site loop)] confine the interface that is localized around twofold symmetry axis. Interestingly, the pyrrolidine rings of conserved proline residues are located at the core of these loops at distances of ∼6 Å and are antiparallel with respect to each other (Meng et al., 2006). Comparison of structures of *Hs*P5CR and *Os*P5CR (residue numbers are given in parentheses; see below) reveals a key set of highly conserved inter-unit salt bridges that stabilize the interface. These are formed by the interaction of R199 (R212) with D229 (D242). Another set of salt bridges is created between D190 (D203) and K228 (K241), and H223 (H236) and D229 (D242), (**Figure 4C**). Additionally, a very interesting π–π stacking interaction between two related R264 (R251) residues from the adjacent protein subunits is present in the middle of the dimer–dimer interface. All of the residues forming these salt bridges appear to be preserved in decameric P5CRs and are conserved in plants representatives displayed in **Figure 2**.

### Can Dimer/Decamer Determinants Be Predicted from the Sequence?

The interfaces in proteins are formed by the interactions of favorable tight fitting regions. These regions are characterized by complementary areas distributed throughout the interface, which are enhanced by the presence of structurally conserved residues (Nooren and Thornton, 2003). These regions are considered “complementary” because they pair both their shapes and the collection of hydrophobic and hydrophilic residues within hydrophobic cores and “hot-spots” of charged residues (Moreira et al., 2007). The structural and sequence comparison of P5CRs shows that their complementary regions are located at the C-terminus, and they are extremely similar (for example in *Os*P5CR, 79 out of 107 residues are conserved or of similar character, **Figure 2**). Additional evidence of the importance of the C-terminal region to decamer formation comes from the analysis of the truncated and full-length forms of *Bc*P5CR. The full-length *Bc*P5CR protein (Bc_2977, 272 residues) has been confirmed to form a decamer under our experimental conditions (Supplementary Figure S1, the experimental *M*w of 275 kDa vs. a deduced theoretical *M*w of 293 kDa). On the contrary, the structure of the truncated *Bc*P5CR (PDB id: 3GT0; residues 1–217, missing the last 55 residues) was calculated by software PISA (Krissinel and Henrick, 2007) to be either a dimer or a tetramer. This strongly suggests that the last 3–4 helices of the dimerization domain (the last 55 residues for a typical-size P5CRs) are important for proper oligomer formation. An inspection of the sequences of selected representatives of P5CRs seems to corroborate this further as significant variation of sequences are observed between selected dimeric (*Neisseria meningitides, Nm*P5CR and *Coxiella burnetii, Cb*P5CR) and decameric members (*Hs*P5CR and *Os*P5CR) at this region. Therefore, we propose that specific sequence differences may provide an explanation why some P5CRs form dimeric structures while others assemble into decamers.

The most noticeable differences are found in the sequences that span between the C-terminus of α8 and the N-terminal part of α9. The sequence motif (residues 202–214 in *Os*P5CR) that is conserved in plant P5CRs and *Hs*P5CR A-D-**G-G-**V-A-A-G-L-**P-**R-D/R-L is replaced by Q-N-**A-A-**I-R-Q-G-F-**D-**M-A-E in *Nm*P5CR (dimer) and Q-E-**A-A-**E-Q-L-G-L-**T**-K**-**E-T in *Cb*P5CR (dimer). Mutation of glycine and proline residues located within this motif (G204, G205, P211 in plant decamers) to following residues (A204, A205, D211/T211 in dimers) likely changes the conformation of this region and disrupts its potential interface surface. In addition, replacement of conserved proline and glycine residues in *Hs*P5CR (P198, P224, G225, and P234) with four different residues (D188, F214, E215, K224 in *Nm*P5CR) and three residues (T198, V224, V225 in *Cb*P5CR) might be even more disruptive. The proline residues are important in establishing complementary “*sharp turns*” of the loops between the dimer–dimer interfaces and bringing both interfaces closer together (**Figure 4**). Also, replacement of proline residues with bulkier residues increases the packing distance between molecules, and likely dislocates the interacting backbones. In addition, some of the residues that were shown to form the conserved salt bridges at the protein–protein interface of the decamer (**Figures 2 and 4C**) are missing in the sequence of dimers. For example, the symmetry related R264 residues were mutated to alanine in *Nm*P5CR and to threonine in *Sp*P5CR, while R212 (*Os*P5CR) was changed to methionine in *Nm*P5CR. Our analysis shows that the dimer–dimer interface network pattern appears to be similar. Sequence comparison suggests that these replacements at the C-terminal region could be a reason, why certain representatives of the P5CR family do not form complementary dimer–dimer interfaces. Detailed biochemical and mutagenesis studies will be required to provide conclusive experimental evidence.

### Sequence Analysis of Dinucleotide Binding Domain (NADPH vs. NADH Preference)

Analysis of the NADH/NADPH binding domains across the P5CR family showed that they can utilize both NADH and NADPH as a reducing agent, while their affinity for either one varies between the species and, sometimes, between different subcellular isoforms. The only difference between these cofactors is the presence of a phosphate group in NADPH, which replaces ribose 2-hydroxyl of adenosine in NADH. The phosphate group in NADPH does not influence the redox abilities of the molecule from the enzymatic standpoint. Both nucleotide cofactor pairs (NADH/NAD^+^ and NADPH/NADP^+^) serve as donors and/or acceptors of reducing equivalents quite efficiently in living cells, and have the same midpoint potential (-0.32 V). However, the additional phosphate group allows enzymes to discriminate between NADH and NADPH, which in turn allows the cell to regulate them both independently.

**FIGURE 5:**
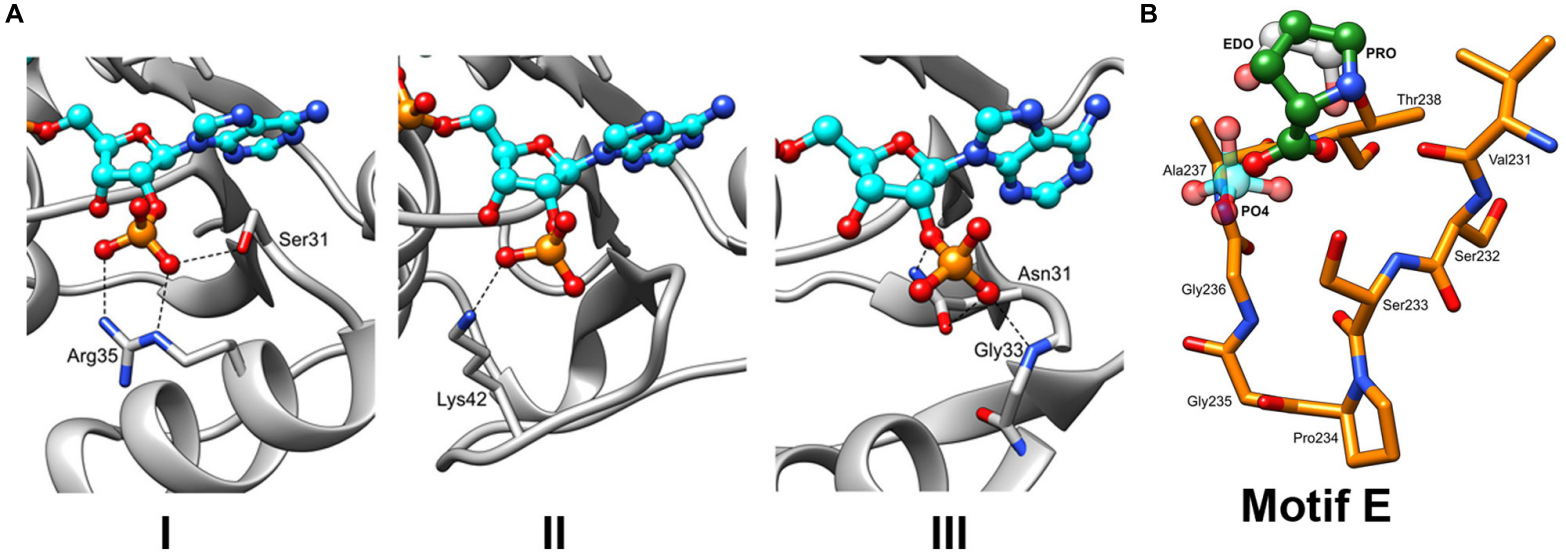
**The insights into the binding of NADPH and L-Proline. (A)** Three different types of interactions with the NADPH phosphate moieties can be hypothesized based on the previously characterized P5CR structures with the following residues: (Type 1) serine (S31) and arginine (R35), as observed in structure of *Sp*P5CR (PDB id: 2AHR). (Type 2) lysine (K42) of *Pf*P5CR (PDB id: 2RCY). (Type 3) Asparagine (N31) of *Nm*P5CR (PDB id: 2AG8). **(B)** Close-up view of the motif E. Structures of enzymes with either buffer-derived molecules (phosphate ion shown in light blue and ethylene glycol molecule shown in gray) or L-proline bound (in green, as observed in the structure of *Sp*P5CR) were superimposed. For clarity, only the structure of *Hs*P5CR is displayed in orange.

In the last 10 years, several complex structures of P5CR with cofactors have been determined, explaining the molecular basis of cofactors binding (Nocek et al., 2005; Meng et al., 2006). Superimposition of the structures of selected representatives of the P5CR family (PDB id: 2RCY, 2IZZ, 2GR9, 2AHR, 2AG8), including the recently determined low-resolution structure of *Os*P5CR (Forlani et al., 2015), showed virtually identical architecture of the NAD(P)H binding domain for all representatives. In addition, it appears that NAD(P)H binding modes are very similar in bacterial and human P5CRs, and is correlated by the high sequence similarity of the regions (Motifs A, B and C) involved in interaction with the cofactor. Therefore, a model of the cofactor binding was predicted and closely correlated with structures/sequences of eukaryotic representatives of P5CR (*Hs*P5CR and *Os*P5CR). Several structures have shown that the cofactor molecule binds in an extended conformation in a cavity between the N-terminal domain and the dimerization domain burying the nicotinamide ring of the cofactor in the active center pocket (Nocek et al., 2005). The cofactor interactions with the protein are characterized by three fingerprint regions, as shown in **Figure 3A** (Motifs A, B, and C). Motif A is a loop formed between the second strand (β2) and the second helix (α2) of the N-terminal domain. It provides positively charged residues for a direct interaction with the adenine moiety, and a 2 phosphate-binding region for NADPH. Three different modes of the NADPH phosphate group interaction can be inferred based on the two bacterial (*Sp*P5CR and *Nm*P5CR, Nocek et al., 2005) and *Plasmodium falciparum* P5CR (*Pf*P5CR) structures, showing high propensity for the interactions involving serine and arginine (mode 1), lysine (mode 2) or asparagine (mode 3) (**Figure 5A**). Most of plant P5CRs has a sequence motif H-R-R-x-x-R (residues 53–58 in *Os*P5CR) or its variation with H-x-N-x-N-R (residues 53–58 in *At*P5CR), (**Figure 2**). The preference for histidine and arginine residues in this region is especially interesting as the side chains of those residues are very often found to form preferable π–π stacking interactions with the adenine ring of the cofactor (Mao et al., 2003; Pyrkov et al., 2009; Firoz et al., 2011). In addition, arginine residues are favorable residues for NADPH binding, and their guanidine side chains have been found to play a key role in binding of the 2′-adenosine phosphate, either alone [as it was observed in the NADPH-*Sp*P5CR complex structure (Nocek et al., 2005)] or in concert with other arginines or lysines (Levy et al., 1996; Sabri et al., 2009). The presence of one or two positively charged amino acids in this region could be a good determinant of the enzyme’s selectivity toward a phosphorylated version of NADH, and mode 1 binding interaction.

Motif B, with the consensus sequence G-x-x-G-x-G-x-M/L (a variant of the typical G-x-G-x-x-G pattern motif of the Rossmann fold) is well conserved throughout the plant P5CRs as it forms a loop that can interact with charged groups of nearby pyrophosphate moieties of either of the cofactors. The glycine-rich loop is placed between the C-terminus of the strand β1 and the N-terminus of helix α1, which forms a dipol and provides charge stabilization of the phosphate group. The positioning of the glycine residues in this region, which due to their lack of the side chains have the lowest steric hindrance, is highly advantageous as it allows for close contact between the pyrophosphate group and the backbone of the adjacent helix. Mutations of glycine residues in the glycine rich region (Motif B) have been reported to reduce or completely eliminate enzyme activity (Wierenga et al., 1985)

There is another set of residues that should be included as a part of the motif B, as it influences the position of the pyrophosphate moiety of the cofactor, even though it is ∼60 residues apart from the glycine-rich signature. This additional set of residues forms a loop positioned between β4 and α4 and encompasses a consensus sequence V–K–P. The conserved lysine residue in the middle of this motif (**Figure 2**), which is present in all representatives of P5CRs, acts as an anchor either directly through the interaction with one of the phosphate groups of the pyrophosphate, or indirectly by reducing the space around the pyrophosphate and pushing it toward the glycine-rich region on the other side of the cavity.

Finally, the last of nucleotide binding elements is motif C. It forms a conserved loop at the active site (132–136 in *Os*P5CR) with the R-x-M-x-N sequence. The methionine residue represents one of several non-polar residues that contribute to the active site pocket and is in proximity to the nicotinamide ring of the cofactor. This and another methionine, which is part of motif B, surround the cofactor’s nicotinamide ring and are major contributors to the hydrophobic environment, which is likely required in the active site to provide the essential hydride transfer step.

### Pyrroline-5-Carboxylate/Proline Binding Site

In plants, sequence alignment of the P5CR family highlights a conserved consensus sequence motif S-P-A/G-G-T-T (Motif E) that is located at the C-terminal part of the protein (**Figure 2**). Very similar motifs are present in other organisms, demonstrating a common structural feature. This motif is located between helices β10 and β11 and creates a tight turn (α-turn), which reverses direction of main chain helices β11–β12, and forms a small cavity (Nocek et al., 2005). Presence of proline (cyclic structure) and glycine (the most accommodating sterically) residues at the center of the cavity is ideally suited for the α-turn as it allows for favorable positioning of serine and threonine at the boundaries of the cavity (**Figure 5B**). The backbones of these non-polar residues were observed to interact with the carboxylate group of L-proline (the product) in the structure of *Sp*P5CR (Nocek et al., 2005), suggesting their essential role in positioning of the substrate in the active site. However, structure with L-P5C (the substrate) has not yet been reported, and it is a prediction based on the current structural results. In addition to binding the product of the enzymatic reaction, the same pocket was observed to bind a phosphate ion (∼2 M potassium phosphate ion concentration was reported in crystallization condition for *Cb*P5CR, PDB header) and 1,2-ethanediol molecule, revealing affinity for small anions.

### Conformational Changes Observed in Selected P5CRs

**FIGURE 6:**
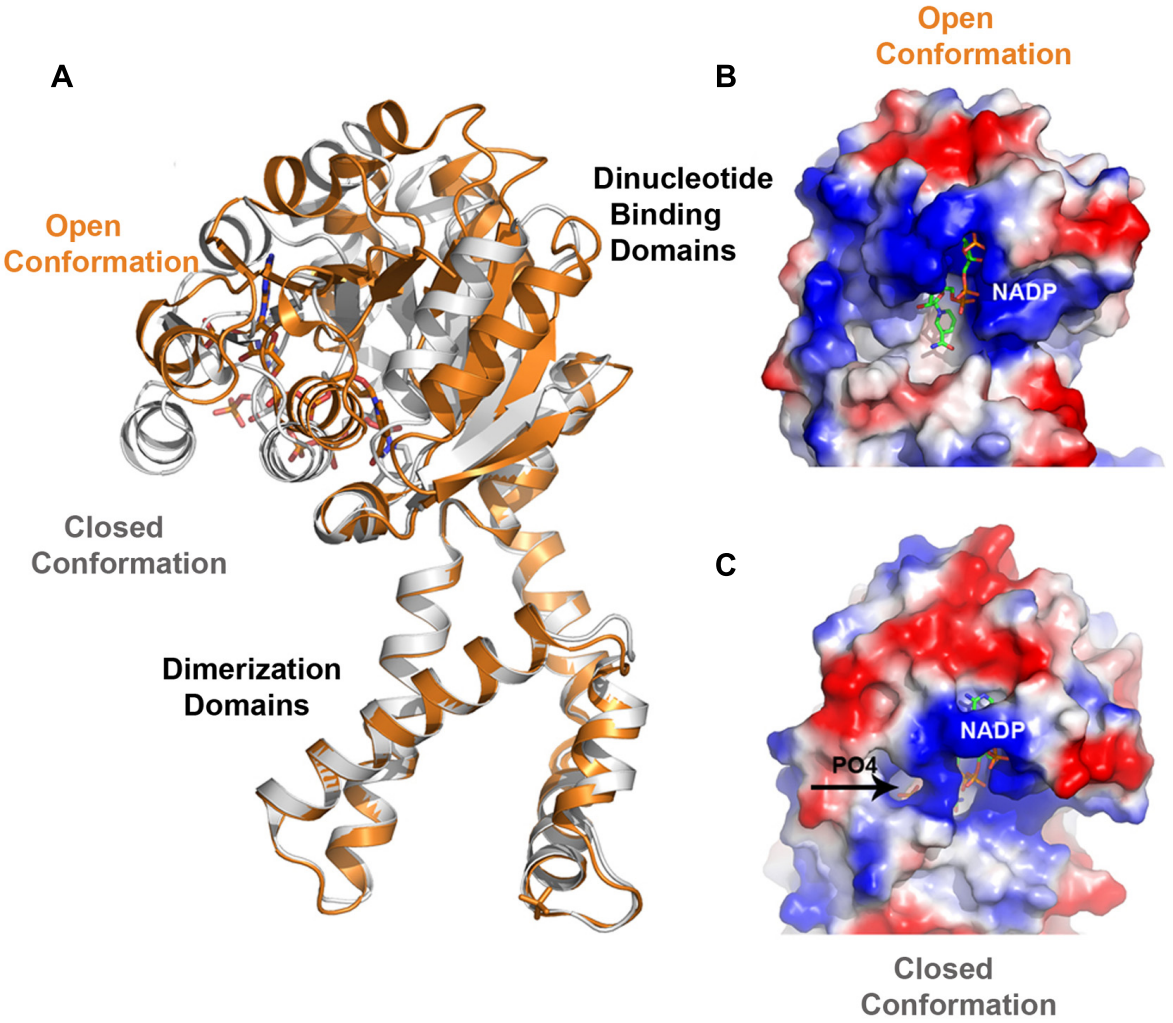
**Superimposition of *Cb*P5CR structure showing differences in the relative orientation of the N- and C-terminal domains and a close-up view of the active site of *Cb*P5CR. (A)** Superimposition of two subunits of dimer in the *Cb*P5CR structure showing differences in the relative orientation of the N-terminal domains (C-terminal domains were optimized) and revealing a large scale of the domain movement. **(B,C)** Surface representation of the active site areas in the two different conformations: **(B)** Open conformation, showing NADP^+^ molecule as green sticks, **(C)** Closed conformation, NADP^+^ and phosphate ion (shown as sticks and indicated by an arrow) are enclosed in the active site.

Pyrroline-5-carboxylate reductase enzymes have their dinucleotide domains loosely connected to the tightly packed dimerization domains *via* a predicted hinge region (Motif D; **Figure 4A**). Hinge regions are often placed between domains and are attributed the role of moderating the conformational movement of the domains. The structural superimposition of monomers of selected P5CRs revealed almost uniform arrangement of subunits in the decameric molecule (root-mean-square deviation (r.m.s.d.) of 0.3-Å in *Sp*P5CR and r.m.s.d. of 0.5-Å in *Hs*P5CR between the most divergent regions). This indicates lack of significant structural changes and minimal differences in the relative orientation of the N- and C-terminal domains. This also allows us to believe that the previously proposed lock-and-key model, and the concept of enzyme undergoing only small rearrangements, is correct for some of the studied enzymes (Nocek et al., 2005). However, more prominent differences were observed in the dimeric structure of *Cb*P5CR (PDB id: 3TRI; Franklin et al., 2015) revealing a substantial r.m.s.d. of 2.5-Å and indicating large changes in the relative orientation of the N- and the C-terminal domains (**Figure 6A**). In fact, pairwise structural alignment of both monomers in *Cb*P5CR (with the C-terminal domains closely aligned) unveils ∼7-Å movement and the difference in the orientation of the respective N-terminal domains. A close examination of domains in the dimer shows that both have a NADPH molecule bound; yet only one of the active sites has a phosphate ion present in the area of the motif E (L-proline binding motif; **Figure 6C**). The active site with both NADPH and the phosphate ion bound adopts a closed conformation, with the dinucleotide positioned in a narrow positively charged cleft and phosphate ion enclosed in the catalytic pocket (closed-conformation). The second active site, with only NADPH molecule bound, is wide and appears to be in an open-conformation (**Figure 6B**). Hence, the only difference between both sites is a presence of the phosphate ion bound in the active site. This suggests that the phosphate ion might trigger conformational changes and possibly acts as a mimic/inhibitor of L-proline/P5C in *Cb*P5CR. It is also important to note that the open-to-closed motion in *Cb*P5CR brings together critical residues from both domains, allowing the formation of a functionally competent active site.

The presence of the dynamic movement of the N-terminal domain and conformational changes in the active site may have their consequences for the mechanism of the ligands binding and the release of the products. In previous studies, the decameric structure of *Sp*P5CR in complex with NADP^+^ revealed that the active site had insufficient opening for L-proline to enter (Nocek et al., 2005). This led to a hypothesis that the order of substrates binding might require L-P5C to bind ahead of the coenzyme, and was later confirmed experimentally (Petrollino and Forlani, 2012). In addition, experimental evidence supporting an ordered substrate binding in plant P5CRs has been obtained from the kinetic analysis of enzyme inhibition by some aminobisphosphonates (Forlani et al., 2007). The *Cb*P5CR structure implies different possibilities in which the NADPH cofactor binds first ahead of the L-pyrroline, or both of them bind simultaneously as the active site seems to be fully open. However binding of one of the substrates triggers conformational changes as it has been observed in case of other NAD(P)H-dependent reductase (Sanli et al., 2003).

It remains to be seen whether this mechanism is universal for other P5CR enzymes or is only limited to *Cb*P5CR and/or reflects flexible nature of some representatives of the P5CR family. On this note, significant dynamic movements of the N-terminal domains have been observed in the *Os*P5CR structure, hindering modeling of these domains in the low-resolution decameric structure, and indicating that dynamic movement is also present in decameric plant P5CRs (Forlani et al., 2015).

### Feedback Inhibition and Metals Effect on Activity of P5CRs

In enzymatic reactions where the product resembles the starting reactant, product inhibition can be often observed. This kind of feedback provides a very efficient mechanism of controlling concentration of the product of the reaction and regulating resources in the cell. The last step of the synthesis of proline, catalyzed by P5CR enzymes, was reported to be generally unaffected by feedback inhibition in plants (Szoke et al., 1992), in contrast to the first enzyme of the glutamate pathway (P5C synthetase; Aral and Kamoun, 1997; Kavi Kishor et al., 2005). However, recent studies on *At*P5CR showed that this enzyme uses both NADPH and NADH, displaying a much higher affinity for the former (Giberti et al., 2014). When NADH was used as the electron donor, feedback inhibition by high yet physiological proline concentrations was reported. Structural studies of bacterial *Sp*P5CR revealed that one of the possible mechanisms by which proline may inhibit P5CR enzymes is by partially blocking the access to the active site (Nocek et al., 2005). Analysis of proline and cofactor-bound structures of *Sp*P5CR showed that one L-proline molecule was bound in the active site (Motif E), but the second L-proline molecule was found in the center of the active site pocket, at the position typically occupied by the nicotinamide ring moiety of the cofactor. It suggests that accumulation of product of the reaction in the active site might prevent NAD(P)H from binding of substrate and inhibit the reaction.

In addition, other studies on P5CRs have shown that metal ions may have either inhibitory or stimulatory effects on the enzymatic activity depending on the nature of the metal and its concentration. For instance, a stimulatory effect of 100 mM KCl or 10 mM MgCl_2_ on the NADH-dependent reaction for partially purified P5CR from *Pisum sativum* L. or *Mycobacterium tuberculosis* P5CR were reported (Rayapati et al., 1989; Yang et al., 2006). As metal binding sites in proteins differ in they coordination numbers and geometries, and their preference for certain environments, therefore sometimes their binding sites could be predicted. For example, the so-called ‘alkali class’ (Ca, K, Na, Mg) consists of metals that interact almost exclusively with oxygen atoms (Zheng et al., 2008). The ligands that often interact with alkaline class ions include side chains of aspartic, glutamic, serine, and threonine residues, or backbone carbonyl oxygen and water molecules. Occasionally, asparagine and glutamine side chains are also found to interact with metals. An inspection of P5CRs structures and sequences showed limited occurrence of the cluster of these residues within the active site and revealed only two potential locations. Close analysis of the *Os*P5CR structure shows that one of them is positioned close to the adenine moiety of the cofactor, and has only three suitable residues that potentially could interact with metal ion (S33, T59, and N56). The second one is the part of motif E (T184, S189, T250, and T251) that is involved in binding of the L-pyrroline mojety. Binding of the metal in the active site would certainly explain the inhibitory effect, however, it remains to be seen if metals could bind there.

## Summary

The P5CR enzymes have been studied for several years, but remain relatively poorly understood from the structural perspective. Particular areas of interest include: structural details of cofactor preference and recognition, substrate binding site, oligomerization and metals effect on activity. The patterns that emerged from this comprehensive phylogenetic analysis suggest that a vertical descent dominated in evolution of P5CR genes, particularly in higher plants and algae. The plant P5CRs appear to be distant from the cyanobacterial and are much closer to the metazoan enzymes. At the sequence level, a close similarity of plant and human P5CRs (∼44% sequence identity between *Hs*P5CR and *Os*P5CR) is somewhat visible revealing a consistent pattern of conserved residues. In contrast, a typical bacterial representative such as *Sp*P5CR shares much lower (∼30%) sequence similarity with *Os*P5CR, and shows reduced sequence conservations especially at the dimerization domain region. Despite evident sequence differences, X-ray crystallographic studies of *Hs*P5CR and *Sp*P5CR enzymes unveiled very similar folds. Similar folds have been also observed in other representatives such as: *Nm*P5CR, *Pf*P5CR, and *Cb*P5CR. Analysis of these structures showed that dimer is the minimal form of P5CR required for activity (*Sp*P5CR), while a decamer is another oligomeric form observed. Whether dimeric or decameric P5CRs exist likely depends on the specific sequence motifs. The presence of the conserved small proline and glycine residues in the loops and turns between the helices α8–α9 and α10–α11 increases the flexibility of the interface region and allows on the tight packing and the formation of the higher oligomers. Also, the preserved patterns of electrostatic interactions are present at the dimer–dimer interfaces of decamers of *Hs*P5CR and *Os*P5CR, likely contributing to the formation of the interface between molecules. The sequence alignment of plant P5CRs showed conservation of several functional motifs involved in the binding of substrates. Three sequence motifs (A, B, and C) are involved in the interaction with both cofactors NADH and NADPH. The presence of one or two positively charged amino acids within the motif A, especially arginine residues, could cause the preference toward the phosphorylated form of the cofactor. L-proline binds within the highly conserved motif E that is located at the C-terminus. This motif utilizes two conserved residues (serine and threonine), to interact with the carboxylate group of L-proline, as it was observed in the structure of bacterial enzymes (*Sp*P5CR). In contrast to the N-terminal parts of the P5CR enzymes (the dinucleotide-binding domains), the C-terminal domains (the dimerization domains) showed a remarkably high level of sequence similarity, especially at the regions predicted to be involved in a decamer formation. This suggests that plant P5CRs (shown in **Figure 2**) form higher oligomers, most likely decamers. This is certainly in agreement with the results of the most recent crystallographic studies of *Os*P5CR, which revealed a decameric arrangement (Forlani et al., 2015). The existence of the decameric structures in many of P5CRs is certainly interesting and brings up a question, what is a functional advantage of such arrangements? One of the possible explanations could be linked to the enzyme’s function in osmotolerance. The ring structure has a structural stability that is required to operate in harsh environments such as high ion concentrations and low water content. Also, in order to control hydration the ring structure minimizes the area exposed to solvent vs. equivalent number of representatives, which would be required to perform the same function.

The modular design of P5CRs, and the presence of the conserved hinge region (Motif D) between domains suggest a dynamic behavior. In fact, the conformational rearrangements were observed in *Cb*P5CR structure. The closed conformation of this enzyme is observed in the case when both NADPH and phosphate ion are bound in the active site, while the open conformation is seen for the site that contains only NADPH. This suggests that the phosphate ion might elicit conformational changes, and may suggest that similar changes occur when P5C binds. It remains to be seen if similar dynamic movement and conformational changes are present in other representatives of the P5CR family.

## Conflict of Interest Statement

The authors declare that the research was conducted in the absence of any commercial or financial relationships that could be construed as a potential conflict of interest.
