# Supplementary material for: Evolution of plant δ1-pyrroline-5-carboxylate reductases from phylogenetic and structural perspectives
Source: Front Plant Sci. 2015 Aug 3;6:567. doi: 10.3389/fpls.2015.00567 (PMC4522605; doi:10.3389/fpls.2015.00567)
Supplement: Supplementary file 2 [file Data_Sheet_1.DOCX]

**Evolution of plant δ^1^-pyrroline-5-carboxylate reductases from phylogenetic and structural perspectives**

**Giuseppe Forlani^1^, Kira S. Makarova^2^, Milosz Ruszkowski^3^, Michele Bertazzini^1^, Boguslaw Nocek^4^***

^1^ Department of Life Science and Biotechnology, University of Ferrara, Italy

^2^ National Center for Biotechnology Information, National Library of Medicine, Bethesda, MD, 20894, USA

^3^ Synchrotron Radiation Research Section, Macromolecular Crystallography Laboratory, National Cancer Institute, Argonne National Laboratory, Argonne, IL, USA

^4^ The Bioscience Division, Argonne National Laboratory, Argonne IL, USA

*** Correspondence:** Boguslaw Nocek, The Bioscience Division, Argonne National Laboratory, 9700 S. Cass Ave., Argonne, Il, 60439, USA. Email: bnocek@anl.gov.

1. **Supplementary Material**

**File 1.** A complete phylogenetic tree of P5CRs in the Newick format.

**Figure S1.** Analysis of the oligomeric state of P5CR proteins by size exclusion chromatography. Chromatogram showing elution of two P5CR proteins (*Bc*P5CR (blue), *At*P5CR (red), from the calibrated column. Since both samples had almost identical values only one of them was shown in the inset (arrow). The inset shows the calibration curve obtained by plotting logMW vs. *K_av_* for the following standard proteins: chymotrypsynogen A (25 kDa; IV); albumin (67 kDa; III), Streptococcus pyogenes *Sp*P5CR (275 kDa; II), and thyroglobulin (669 kDa; I)*.*
