# Supplementary figures and images for: Evolution of plant δ1-pyrroline-5-carboxylate reductases from phylogenetic and structural perspectives
Source: Front Plant Sci. 2015 Aug 3;6:567. doi: 10.3389/fpls.2015.00567 (PMC4522605; doi:10.3389/fpls.2015.00567)

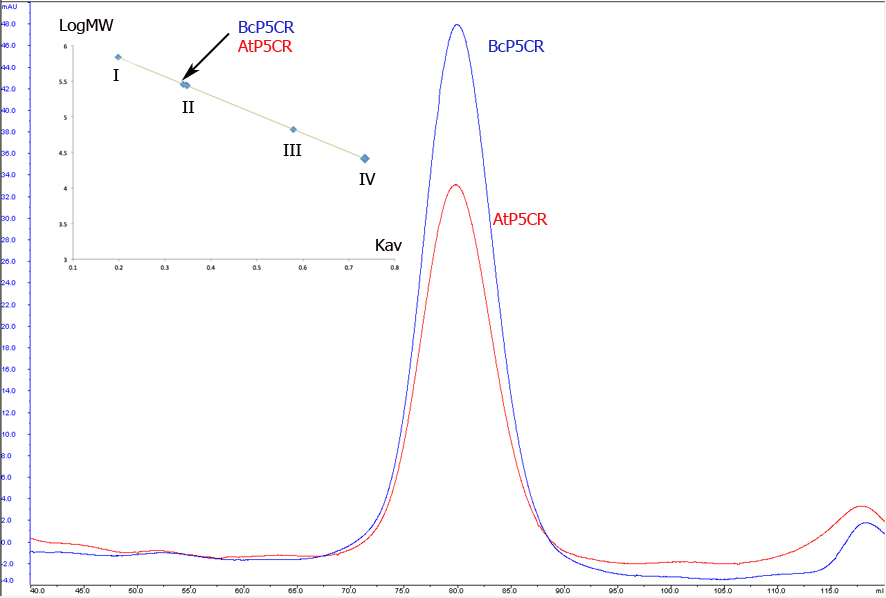

Supplement: Supplementary file 3 [file Image_1.JPEG]
